# Evolution of sociality by natural selection on variances in reproductive fitness: evidence from a social bee

**DOI:** 10.1186/1471-2148-7-153

**Published:** 2007-08-29

**Authors:** Mark I Stevens, Katja Hogendoorn, Michael P Schwarz

**Affiliations:** 1 School of Biological Sciences, Flinders University, GPO Box 2100, Adelaide, SA 5001, Australia; 2 Allan Wilson Centre for Molecular Ecology and Evolution, Massey University, Private Bag 11-222, Palmerston North, New Zealand and School of Biological Sciences, Monash University, Clayton 3800, Victoria, Australia; 3 The University of Adelaide, School of Agriculture, Food and Wine, Waite Campus, Main Building, Adelaide SA 5005, Australia

## Abstract

**Background:**

The Central Limit Theorem (CLT) is a statistical principle that states that as the number of repeated samples from any population increase, the variance among sample means will decrease and means will become more normally distributed. It has been conjectured that the CLT has the potential to provide benefits for group living in some animals via greater predictability in food acquisition, if the number of foraging bouts increases with group size. The potential existence of benefits for group living derived from a purely statistical principle is highly intriguing and it has implications for the origins of sociality.

**Results:**

Here we show that in a social allodapine bee the relationship between cumulative food acquisition (measured as total brood weight) and colony size accords with the CLT. We show that deviations from expected food income decrease with group size, and that brood weights become more normally distributed both over time and with increasing colony size, as predicted by the CLT. Larger colonies are better able to match egg production to expected food intake, and better able to avoid costs associated with producing more brood than can be reared while reducing the risk of under-exploiting the food resources that may be available.

**Conclusion:**

These benefits to group living derive from a purely statistical principle, rather than from ecological, ergonomic or genetic factors, and could apply to a wide variety of species. This in turn suggests that the CLT may provide benefits at the early evolutionary stages of sociality and that evolution of group size could result from selection on variances in reproductive fitness. In addition, they may help explain why sociality has evolved in some groups and not others.

## Background

The Central Limit Theorem (CLT) states that variance among sample means is inversely related to sample size. Furthermore, as sample sizes increase, sample means become more normally distributed, even if the underlying probability density function is asymmetrical or deviates from normality in other ways. Wenzel and Pickering [1] suggested that the CLT entails benefits for sociality in that larger colonies are able to better predict future food acquisition. For example, consider a species where food brought back to the group by foragers is shared among immature offspring. Each foraging trip can be regarded as a stochastic sample of the environment, with the number of foraging trips per unit time corresponding to sample size [1]. Larger group sizes could enable the number of foraging trips per unit time to increase, either because the number of foragers increases, or because group living allows dedicated foragers that are not required to spend time and effort on other nest related tasks (such as cleaning, brood tending, and nest defence) to make more trips. In such cases, a greater number of foraging trips should lead to lower variation among colonies in foraging yield [1]. Consequently, larger groups should produce clutch sizes that match future food acquisition and therefore run a lower risk of either under- or over-producing eggs relative to future resources. If future food acquisition is unpredictable, colonies may either produce more eggs than can be fed and then having to abort some (a 'no wasted food' strategy), or they may under-produce eggs and be unable to fully exploit all the resources that may become available (a 'no wasted brood' strategy) [1].

In addition to the arguments put forward by Wenzel and Pickering [1], Gillespie [2] argued that variance in offspring number can be as important as the mean number of offspring produced when calculating the fitness of alternative genotypes and this holds when variance is due to both environmental and developmental sources of stochasticity. Gillespie showed that selection will favour lower variance in offspring number and the strength of selection will be equal to that on mean numbers in the case of environmental fluctuations. This suggests a very large effect of group size if increasing the group size lowers the variation in offspring production.

The idea that group living may entail benefits because of a purely statistical principle is intriguing as it adds a completely new light to the evolution of sociality. Traditionally, studies looking at the advantages of sociality have focused on benefits deriving from ecological, ergonomic or genetic factors [3-9]. Wenzel and Pickering [1] found a lower percentage of brood abortion events in larger colonies, but did not directly address the variance in brood numbers in relation to the number of females present. Since then there have only been two studies based on a social wasp (*Ropalidia marginata*) that have explicitly examined the CLT in relationship to group living [10,11]. Although Shakarad and Gadagkar [10] established a decreasing variance in brood production with increasing colony size, they were not able to distinguish between benefits derived as a result of the CLT or from a better prevention of brood loss through protection from predation. Naug and Wenzel [11] used behavioural observations from five colonies of the same species and showed that an increase in predictability of foraging success (food supply to the nest) was consistent with the CLT predictions. It appears from these studies that, at least for *R. marginata*, assessing predictability in reproductive success (directly or indirectly) has been problematic.

**Figure 1:**
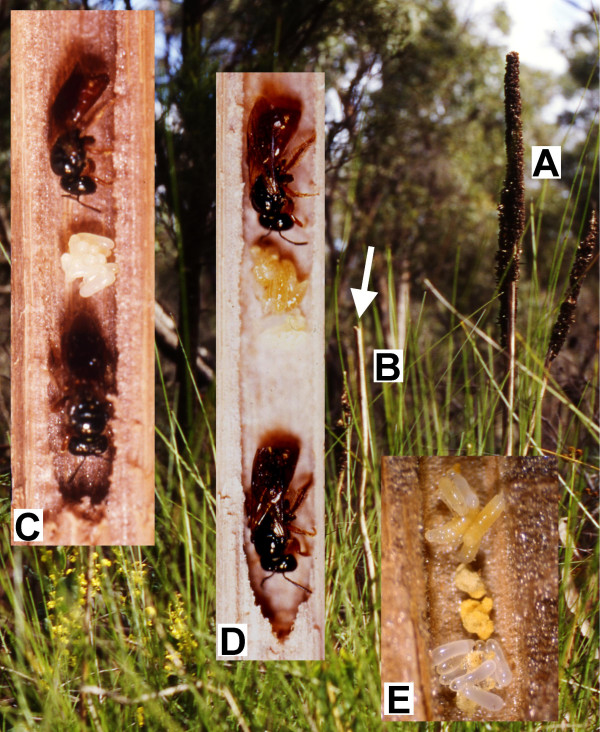
***Exoneura *rear their brood progressively in a communal undivided burrow, and food resources are shared among developing brood**. (A) *Xanthorrhoea minor *flower scape with seed head; (B) *X. minor *flower scape without seed head, *Exoneura nigrescens *females burrow into the pithy interior (white arrow shows nest entrance) of scapes that have lost their seed heads; (C) Two *E. nigrescens *females at the bottom of the nest with a stockpile of eggs (early spring, before foraging and larval rearing has commenced); (D) Two *E. nigrescens *females at the bottom of the nest with larvae (late spring to early summer) when foraging has commenced; (E) Eggs and larvae (feeding on the yellow pollen, which also gives the larvae their yellow colour) at the bottom of a nest.

Here, we use a univoltine allodapine bee, *Exoneura nigrescens*, until 1998 known as *E. bicolor *from heathland areas, to assess the possible consequences of the CLT for group living, and to examine whether brood rearing patterns are consistent with expectations from such effects. *Exoneura *rear their brood progressively in a communal undivided burrow (see Figure 1A–D), and food resources are shared among developing brood (Figure 1D,E) [12,13]. Therefore, this species shows a much stronger relationship between food acquisition and brood mass than most other social insects where substantial investment in nest enlargement and cell construction is concurrent with brood rearing. This provides a unique opportunity to test whether variance in brood mass decreases, and predictability of brood mass increases, with group size, as was suggested by Wenzel and Pickering [1].

Our approach is two-pronged. Firstly, we investigate the CLT-derived predictions that total brood weight will show less variation and become more normally distributed as colony size increases. These patterns are not predicted by ecological or ergonomic models, which instead predict that brood rearing becomes more efficient or that brood predation is decreased as colonies become larger. Secondly, we investigate whether initial clutch sizes correspond better with final brood sizes for larger colonies than for smaller colonies.

## Results

**Figure 2:**
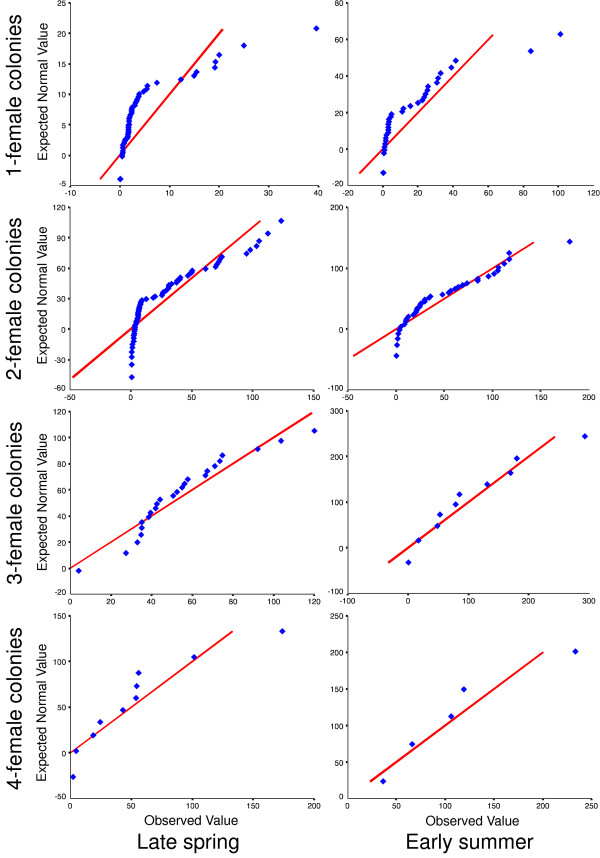
**Normal quantile (Q-Q) plots of total brood weight for 1, 2, 3 and 4-female colonies from late spring and early summer**. Significant deviations from normality (estimated quantiles from an expected normal distribution (straight line) and from the observed values) were found for the distribution of brood weight in 1 and 2-female colonies, but not for larger colony sizes (Table 1).

For our species, the CLT predicts that deviations from mean brood weight should decrease with increasing numbers of foraging trips, and therefore with both time and with number of foragers in the colony (colony size). At the same time the distribution of brood weights should become more normally distributed over time and with colony size. We tested these predictions using total brood weights from the late spring and early summer samples when brood rearing was well underway but brood were not yet mature – *Exoneura *are progressive provisioners (Figure 1) and larval defecation does not occur until shortly before pupation, so that total brood weight reflects the amount of food brought into the colony up until the time of sampling. Earlier samples were not used because most eggs had not yet hatched. Normal quantile (Q-Q) plots (Figure 2) and Shapiro-Wilk tests (Table 1) were used to examine departures from normality for colonies with 1 to 4 females; larger colonies were not tested because of very small sample sizes. For the earlier (late spring) sample, tests showed that the data were not distributed normally for 1 and 2-female colonies, deviated less from normality in 3-female colonies, and were marginally non-significantly different from normality for 4-female colonies (Table 1). For the later (early summer) sample, 1 and 2-female colonies deviated from normality, but 3 and 4-female colonies did not. Thus, colonies with an increasing number of females showed an increasingly normal distribution and later colonies were always more normally distributed than earlier colonies with the same number of females, concordant with predictions from the CLT.

**Table 1:** Shapiro-Wilk tests for deviation from normality in the distribution of total brood weights for different colony sizes in the late spring (6 November) and early summer (4 December) samples

Date	Colony size (females/nest)	Sample size (number of colonies)	Shapiro-Wilk statistic	Significance	Re-sample comparison (Sig.)
November	1	46	0.622	<0.001	-
	2	63	0.810	<0.001	<0.01
	3	25	0.915	0.039	<0.01
	4	10	0.846	0.053	0.08
December	1	31	0.728	<0.001	-
	2	38	0.895	0.002	<0.01
	3	9	0.918	0.375	0.04
	4	5	0.912	0.481	0.19

The re-sample comparison gives the proportion of re-sampled 1-female colony brood weights with higher Shapiro-Wilk values than for 2, 3 or 4-female colonies. These proportions approximate the likelihood that a higher Shapiro-Wilk value could have been obtained from 1-female nests given the same sample size as for the multi-female colony size (see Methods).

Given the widely varying sample sizes for different-sized colonies, we used a re-sampling procedure to ask whether colony-size variation in Shapiro-Wilk values (Table 1) may have been attributable to differences in sample sizes, rather than any underlying differences in normality. The proportion of re-sampled 1-female colony brood weight data with a higher Shapiro-Wilk value than the corresponding multi-female sample approximates the likelihood that the multi-female data is more normally distributed than the 1-female data, and is given in Table 1 for each colony size and sample date. The only cases where these proportions were not less than 5% (i.e. *P *< 0.05) involved 4-female colonies. However, these involved samples sizes of only 10 and 5, for late spring and early summer respectively, where the power of our re-sampling technique is likely to be low. Consequently, our analyses indicate that variation in measures of normality in brood weights is not simply due to variation in sample sizes.

**Figure 3:**
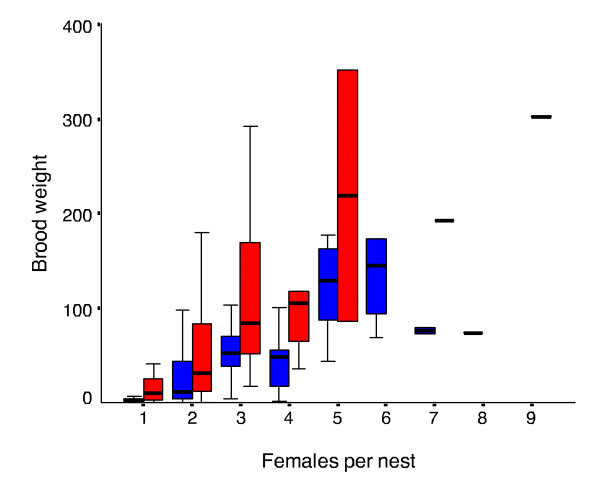
**Boxplot of total brood weight as a function of colony size**. Blue bars (left of number) indicate late spring (6 November) nests and the red bars (right) early summer (4 December) nests. The line shows the median, boxes and the whiskers represent interquartile ranges.

Assessing whether variation in total brood weight decreases with colony size is not straightforward, since total brood weight increases with colony size (Figure 3) and this means that variance in brood weight is likely to vary with mean brood weight as a function of colony size. An ANCOVA showed a significant effect of mean brood weight on the standard deviation in brood weight (*F*_1,8 _= 33.09, *P *< 0.001) (with the standard deviation increasing with the mean), and a significant interaction between mean brood weight and sample date (*F*_1,8 _= 10.64, *P *< 0.05), implying that the coefficient of variation (CV) would be more appropriate as it is independent of the unit of measure.

**Figure 4:**
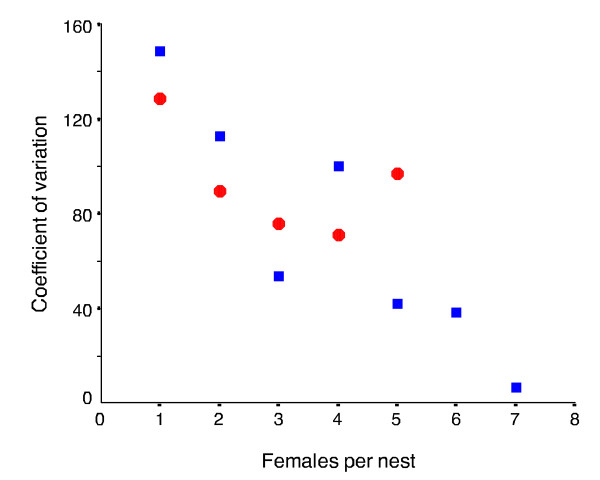
**Coefficient of variation (CV) of total brood weight versus the number of females per nest**. Coefficient of variation (CV) corrected for small sample sizes of total brood weight versus the number of females per nest for the late spring (blue squares) and early summer (red circles) samples. There is a significant decrease in CV with increasing colony size.

The CV is summarised for both late spring and early summer samples in Figure 4. We analysed the correlation between CV and colony size using ANCOVA and the Fligner-Killeen test. We used ANCOVA to investigate whether the CV varied with colony size, taking sample time into account. We tested whether CV for 1-female colonies differed from both 2-female and 3-female colonies using the Fligner-Killeen test (other colony sizes were precluded due to sample size). ANCOVA, with the CV as dependent variable, sample date as a random factor, and colony size as a covariate, showed no interaction between covariate and sample date (*F*_1,8 _= 2.33, *P *= 0.165), but a highly significant effect of colony size (*F*_1,8 _= 12.38, *P *= 0.008). Figure 4 shows a clear decrease in variation with increasing colony sizes, as predicted by the CLT. The Fligner-Killeen test for the late spring sample showed that there was a significant difference between 1 and 2-female colonies (*z *= 2.28, *P *= 0.01), and between 1 and 3-female colonies (*z *= 5.62, *P *< 0.001). However, for the early summer sample, the Fligner-Killeen test indicated no significant differences in CV between 1 and 2-female colonies (*z *= 1.379, *P *= 0.084) or between 1 and 3-female colonies (*z *= 0.271, *P *= 0.394). This latter significance needs to be treated cautiously since the smaller sample size of nine nests for 3-female colonies is close to the minimum sample size of seven recommended for the Fligner-Killeen test [14].

In our second approach, we used ANCOVA with standard deviation as the dependent variable, sample date as the treatment, and mean brood weight and colony size as covariates. Variances were not heteroscedastic (Levene's test, *P *= 0.388), and there was a significant effect of colony size (F_1,8 _= 6.891, *P *= 0.03) and mean brood weight (F_1,8 _= 34.586, *P *< 0.001), but no effect of sample date (F_1,8 _= 0.113, *P *= 0.746). We then pooled the two samples and regressed the standard deviation of brood weight for each colony size-sample date combination onto mean brood weight (F_1,10 _= 25.387, *P *= 0.001, β = 0.847) and saved the residuals. These residuals were then regressed onto colony size with results indicating a significant negative relationship (F_1,10 _= 5.575, *P *= 0.040, β = -0.598). The magnitude of β here suggests a substantial negative effect of colony size on the standard deviation once the effects of mean brood weight are taken into account, while the positive β value for the initial regression indicates that the standard deviation increases with mean brood weight, as expected.

**Figure 5:**
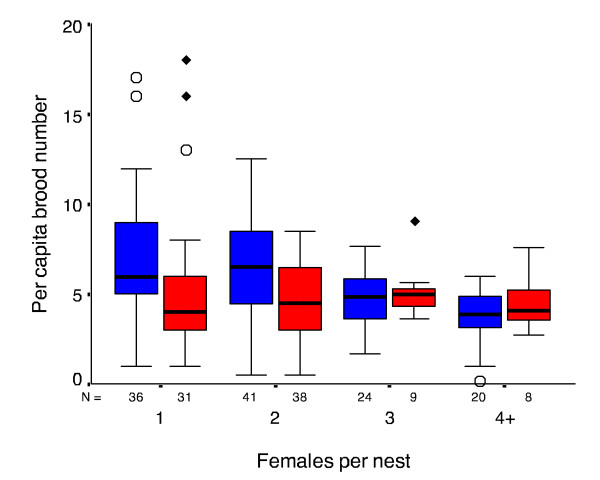
**Boxplots of per capita brood number for early spring samples (blue bars) and early summer samples (red bars)**. Colonies with four or more females were pooled because of low sample sizes. Extremes are indicated by ◆ and outliers are indicated by ○ The line shows the median, and boxes and the whiskers represent interquartile ranges. Sample sizes are indicated directly below the abscissa.

We then investigated the correspondence between initial clutch size in early spring and number of offspring present in summer for different sized colonies using per capita brood numbers from the first sample (early spring) when egg-laying was well underway but larval rearing had only just commenced (Figure 1C,D), and the last sample (early summer) when the oldest brood were nearing maturity. The mean per capita number of brood significantly differed with colony size in spring (Kruskal-Wallis test, χ_3_^2 ^= 22.39, *P *= 0.001), but this was no longer the case by early summer when brood were more mature (χ_3_^2 ^= 2.175, *P *= 0.825). We then explored how these seasonal differences in per capita brood numbers arose by comparing spring and summer per capita values for different colony sizes. Figure 5 shows per capita number of brood for spring and summer samples as a function of colony size, where colonies with four or more adult females are combined because of small sample sizes. For 1 and 2-female colonies, per capita brood numbers were lower in summer than in spring, whereas spring and summer values closely matched for larger colonies. This indicates the occurrence of brood reduction in smaller but not larger colonies.

Consequently, we further assessed this statistically by comparing per capita brood numbers for each colony size category between spring and summer samples using Mann-Whitney U-tests, restricting analyses to colonies with four or fewer females because of small sample sizes for larger nests. Because we used a non-parametric test for comparisons between spring and summer colonies with the same number of adults, these tests also correspond to comparing total brood size. Brood numbers significantly declined between spring and summer for 1-female colonies (Mann-Whitney U test Z = -2.338, *P *= 0.019, asymptotic 2-tailed significance) and 2-female colonies (Z = -2.561, *P *= 0.010), but not for 3-female (Z = -0.223, *P *= 0.823) or 4-female colonies (Z = -1.467, *P *= 0.149). Thus, in small colonies the number of offspring being reared by summer was significantly smaller than the initial clutch size, indicating that brood had been reduced, while for large colonies final offspring numbers corresponded with initial clutch size.

## Discussion

Our study provides several lines of evidence for the existence of benefits that derive from the CLT, rather than from ecological, ergonomic, or genetic factors. Firstly, we have shown that colonies with an increasing number of females showed an increasingly normal distribution of brood weight. For colonies with the same number of females, the brood weight became more normally distributed as the season progressed (Figure 2; Table 1). Secondly, we have shown a clear decrease in variation in total brood weight with increasing colony sizes (Figure 3). Interestingly, the decrease in CV with increasing colony size appears to be lower for the early summer sample (Figure 4 and Fligner-Killeen tests). This is not surprising, since by early summer all colony size categories would have experienced a greater number of foraging trips than in late spring, and under the CLT this should lead to greater normality in the distribution of total food income.

The mean per capita number of brood differed with colony size early in the season, and was higher for small colonies than for larger colonies. By early summer, when brood was more mature, this was no longer the case (Figure 5). This suggests that some small colonies may be initially over-producing brood, a 'no wasted food' strategy. In contrast, larger colonies appear better able to predict future food income and final clutch sizes and therefore better able to avoid/lessen the costs of aborting excess brood in the event of low food income, a 'no wasted brood' strategy.

The benefits that the CLT might provide to larger colonies of *E. nigrescens *cannot be quantified from our data because we do not know the costs involved in abortion of brood, but these are likely to be substantial. Brood are reared progressively, so that resources are sequestered in the developing brood over time, and if not all brood can be reared then already-invested resources will be lost. Oophagy and larval cannibalism in *E. nigrescens *are extremely rare [15] so it is unlikely that investment in aborted eggs and larvae is recovered to any large extent. Importantly, egg size in allodapines is very large (egg length is approximately 30% of total body length, see Figure 1C) compared to most other social insects [16], so that over-production of eggs is likely to be costly, and abortion of larvae, where even further resources have been invested, will be more so. Therefore, colonies that can better match brood numbers to food acquisition should have a selective advantage. Such benefits derived from the CLT clearly refute Alexander's [17] assertion that there are three automatic and universal costs to group living but no universal benefit.

Even if colonies are not able to benefit from better matching egg production to expected food income, the lower variation in mean brood weight should provide benefits via the arguments suggested by Gillespie [2] for selection on lower variance in offspring numbers. The arguments put forward by Wenzel and Pickering [1], and supported by our results, suggest that lower variation in brood weight is due to the effect of group size on variation in food acquisition, ultimately deriving from stochasticity in the environment. Gillespie's [2] models suggest that the selective effects of this may be equivalent to changes in mean brood numbers.

Group living, especially where it involves behavioural specialisation, is likely to provide benefits from many sources, such as enhanced task efficiency [3,4,9,18-20] and protection against predators [21-23]. However, the CLT has the potential to enable benefits for group living without involving any behavioural specialisation and requires only that larger groups have higher numbers of foraging trips and that the incoming food is shared by the group [1,11]. Indeed, Naug and Wenzel [11] demonstrated that an increase in predictability of foraging success (food supply to the nest) in the social wasp *R. marginata *was consistent with the CLT predictions. Such basic requirements suggest that the CLT may provide some benefits at the early evolutionary stages of sociality, and that selection can act on variances in reproductive fitness [see [2]].

Given the potential for the CLT to provide benefits to group living, it is surprising that more studies have not assessed it as an explicit factor shaping group performance as a function of group size. However, increased reliability and predictability in offspring production has recently been suggested as a factor influencing colonial nesting in birds [24], and may be important in shaping the group size of communally breeding birds. Reed and Walters [25] investigated the effect of the number of helpers on variance in reproductive success in Red-cockaded Woodpeckers (*Picoides borealis*) and, using the literature available, in other communally breeding birds. They found that the presence of helpers was often associated with higher rather than lower variances, but pointed out that this result could have been confounded by effects of habitat quality, a factor that was ruled out in our experimental set up [15]. In contrast, data presented by Woxvold [26] suggest a lower variance with increasing group size for apostle birds (*Struthidea cinerea*), emphasizing that breeding systems, among other factors, need to be considered.

## Conclusion

There are likely to be a large number of factors that influence when and how group living evolves [e.g. [5,20,27]], but the non-random distribution of social origins with respect to life-history traits [28] suggest that some factors are more important than others. The ability to pool group resources when provisioning offspring means that foraging bouts, which represent samples from the environment, increase with group size. The importance of large sample sizes are well known to biologists – they increase our ability to more accurately estimate population parameters, and ultimately this ability derives from the CLT. It would be surprising if this statistical property has been unexploited during the tinkerings of natural selection. The importance of CLT needs to be further explored in other social groups to determine its wider potential facilitating role in social evolution.

## Methods

### Location and data collection

The study was carried out in Cobboboonee State Forest, Victoria, Australia (38°77'S, 141°35'E). Occupied nests (*Xanthorrhea minor *flower scapes, Figure 1B) were collected from an area approximately 400 m by 2000 m on 31 July 1996 [15], when colonies were overwintering and before egg production had begun. We placed equal numbers of nests (randomly allocated) in four nearby sites [15]. Over three collecting trips (21 September (early spring), 6 November (late spring), 4 December (early summer) 1996) an equal number of *E. nigrescens *nests were collected from the four sites [15]. Nests were stored at 10°C and each collection processed within two days. For each colony we obtained a weight (mg) for the total number of eggs and for the total number of larvae. Because the number of adult females also relates to the possible information gathered about future food acquisition, irrespective of who is reproductive at any one time [15], we define brood per capita as 'the number of brood relative to the number of adult females', which was calculated by dividing the total brood (eggs, small larvae, medium larvae, large larvae, pre-pupae, and pupae) in the nest by the number of adult females in the nest. For each site and field collection, the mean and standard deviations in the number of brood per capita and brood weight were calculated for 1-female, 2-female, 3-female, and 4 (or more)-female colonies.

### Data analyses

Data were analysed with SPSS version 11 for Macintosh. Where assumptions of homoscedasticity could not be met and where any variable was ordinal or categorical, non-parametric tests were used. Previous studies of *Exoneura *show that per capita brood number increases with colony size [22,29,30] and this is largely due to avoidance of total brood loss to enemies-at-the-nest [31] – allodapine brood are not enclosed within cells (see Figure 1C–E) and are therefore highly vulnerable to predation [22]. To avoid this confounding ecological benefit of group living, we restricted our analyses to nests with brood. The nest contents did not differ between sites for any of the sampling dates (Kruskal-Wallis 1-way ANOVA, all *P *> 0.05) [15] and were thus pooled into one collection per sampling date.

We assessed normality of total brood weights for 1, 2, 3 and 4-female colonies using quantile-quantile (Q-Q) plots and Shapiro-Wilk tests. However, the power of such tests may vary with sample sizes, and in our case these varied widely with colony size. In order to examine whether sample size was sufficient to explain the outcome of the Shapiro-Wilk tests, we used a re-sampling procedure, which compared measures of normality for total brood weight in 1-female colonies with 2, 3 and 4-female colonies, larger colonies were not used because they were too few for meaningful comparison. We re-sampled, with replacement, total brood weights from 1-female colonies using the same sample sizes that we had for colonies with 2, 3 or 4 females (Table 1). Colonies with 2 females were more common than 1-female colonies (Table 1), so for these we randomly selected a subset of the 2-female colonies to produce the same sample size as for 1-female colonies and calculated the Shapiro-Wilk values for these subsets. Re-sampling with replacement asks whether an underlying distribution of brood weights for 1-female colonies are likely to yield more-normal distributions for 2, 3 or 4-female colonies for each of the respective sample sizes. For each protocol, we used 100 random re-samples and determined the proportion of cases that had higher values of the Shapiro-Wilk statistic than was calculated for the multi-female colonies. This proportion represents a one-tailed significance test because in each case we explicitly asked whether multi-female colonies had more normally distributed brood weights than 1-female colonies.

Variation around sample means can be estimated using the standard deviation but interpretation of the SD can be problematic if variation increases with the mean, a common phenomenon [32]. In such cases the coefficient of variation (CV) is a more useful tool [10,11,32]. The CV is independent of the unit of measure, and is the standard deviations (SD) expressed as a proportion of the means (y), so CV = SD/y, often converted to be a percentage of the mean; but here we use it as a proportion. We corrected for small sample sizes by multiplying the CV by (1 + 1/4n) [32]. However, calculating the CV without assessing if the standard deviations are affected by the mean may result in an over-compensation and produce false trends [32]. Therefore, the relationship between the mean and SD of brood weight was analysed using an ANCOVA.

Comparing CV across different groups is not straightforward, and most existing methods have high Type I error rates and are not robust to differences in underlying distributions, such as differences in kurtosis or skewness [14]. The modified Fligner-Killeen test [14] suffers least from these drawbacks but is designed for only pairwise comparisons and requires that the smallest sample size for any comparison be ≥7 [14]. We used the Fligner-Killeen test to compare CV for 1-female colonies with colonies of 2 and 3-females. Small sample sizes precluded comparisons between colonies in the other size categories.

As a further check on results from analysis of the CV, we examined the SD itself. For this procedure we calculated the SD of total brood weight for each colony size for each sample date, and then regressed the SD onto mean brood weight, taking sample dates into account. Residuals were saved and then regressed onto colony size to determine if colony size contributed to the SD independently of mean brood weight.

### Assumptions

We apply several biologically realistic assumptions here, namely that (1) brood weight is a proxy for foraging effort; (2) the existence of differing male and female metabolic rates do not affect our data interpretation; and (3) different metabolic rates do not influence normality of brood weights (increasing normality in larger colonies and over time). These assumptions hold for several reasons [see [33-37]]. Most importantly, if sex ratios are constant for different sized colonies, this would be equivalent to changing the total weight of each colony by a set amount and this would not affect our analyses since we used the CV (see above), which removes the effect of mean colony brood weights. Moreover, because sex ratios vary strongly with colony size in *E. nigrescens *[15,35,36] gender differences in metabolic rates will result in a greater variance in brood weights for larger colonies where there is the possibility for greater variation in sex ratios. Because larger colonies have a much greater incidence of male immatures, then there would be a greater effect of differential metabolic rates, leading to a greater range in individual larval weights and hence a more platykurtic distribution of colony brood weights. However, because 1-female colonies have in most cases only females [36], the distribution of brood weights should tend towards normality.

## Authors' contributions

MPS conceived the study that formed part of MS's honours degree. MS wrote the first draft of the manuscript and performed the initial analyses, while MPS performed the re-sampling and final analyses. All authors contributed to the analysis of the results and to the writing of the paper. All authors read and approved the final manuscript.
